# Knowledge and Attitude of Nurses in Wuxi, China Towards Alzheimer's Disease: A Cross‐Sectional Study

**DOI:** 10.1002/nop2.70199

**Published:** 2025-04-01

**Authors:** Rong Wang, Yunyun Gu, Huan Yang, Song Ge, Yinghua Cai, Xia Wan

**Affiliations:** ^1^ Department of Nursing, The Affiliated Wuxi People's Hospital of Nanjing Medical University, Wuxi Medical Center Nanjing Medical University, Wuxi People's Hospital Wuxi Jiangsu China; ^2^ Department of Nursing Wuxi Hospital of Traditional Chinese Medicine Wuxi Jiangsu China; ^3^ Department of Natural Sciences University of Houston‐Downtown Houston Texas USA

**Keywords:** Alzheimer's disease, attitude, cross‐sectional survey, dementia, influencing factors, knowledge, nursing staff

## Abstract

**Aim:**

This study investigated knowledge and attitude towards Alzheimer's disease among nurses in China.

**Design:**

A cross‐sectional design.

**Methods:**

A total of 186 nurses were recruited in Wuxi, China from January to March 2024. The Alzheimer's Disease Knowledge Scale and Dementia Attitudes Scale were used to evaluate nurses' knowledge and attitudes towards Alzheimer's disease. Descriptive statistics, univariate analysis and multivariate linear regression analysis were utilised to evaluate the levels and influencing factors of their knowledge and attitudes towards Alzheimer's disease.

**Results:**

The participants' average the Alzheimer's Disease Knowledge Scale and Dementia Attitudes Scale scores were 20.82 ± 2.31 (approximately 69.4% correctly) and 86.23 ± 14.14 (approximately 61.6%), respectively. Professional titles and whether or not they had received previous training about Alzheimer's Disease were among the factors affecting their dementia knowledge. Education background and workplace were the influencing factors of nurses' attitude towards dementia.

**Conclusion:**

Nursing personnel in the Wuxi region demonstrate a limited understanding of Alzheimer's disease, coupled with positive attitudes towards its care. It is imperative to implement effective strategies to enhance in‐service training for nurses regarding dementia. This initiative aims to elevate their level of knowledge, improve their attitudes towards dementia care, and ultimately enhance the quality of nursing provided to individuals with dementia.

## 1 Introduction

Alzheimer's Disease (ad) is a degenerative neurological condition characterised by progressive cognitive decline, including memory loss and deterioration of mental function, ultimately impairing patients' cognitive abilities, behaviour and daily functioning (Porsteinsson et al. 2021). The quality of care provided to individuals with ad greatly affects their overall quality of life and ability to perform daily tasks, with nursing skills, empathy and attitudes playing a pivotal role (de Witt and Ploeg 2016; Schneider et al. 2020). Prior research has indicated that inadequate ad knowledge and attitudes among healthcare providers can lead to missed and delayed patient diagnoses and timely delivery of quality care (Aminzadeh et al. 2012). However, limited studies have focused on nurses' ad knowledge and attitudes in mainland China (Yaghmour 2022). The lack of sufficient knowledge about Alzheimer's disease among nursing staff in China affects the quality of care provided (Rice et al. 2019; Yaghmour 2022). This study aims to provide insights to develop targeted educational and training strategies.

## 2 Background

Alzheimer's disease (ad) accounts for 60%–80% of all dementia cases, and its high prevalence and severity among the elderly population have garnered global attention. Globally, 55.2 million people live with dementia, and by 2050, this number is expected to reach 139 million (Organization, W. H. 2021). China has the highest number of dementia patients and the fastest growth rate in the world, accounting for about a quarter of the total (Jia, Quan, et al. 2020). A recent study shows that 15.07 million elderly people in China suffer from dementia, including 9.83 million with Alzheimer's disease (Jia, Du, et al. 2020). There is no doubt that the healthcare systems in most countries, including China, are challenged by the management of dementia.

Nurses involved in dementia shoulder diverse responsibilities, including providing direct patient care, supporting families and collaborating with other healthcare professionals. Research has highlighted the substantial impact of primary care nursing staff in dementia care, particularly in performing assessments, making referrals and educating family caregivers (Gibson et al. 2021; Lindauer et al. 2021). Hallberg et al. (2013) analysed dementia care services in eight European countries and found that nurses play a crucial role in a range of support services, including screening, diagnostic procedures, treatment of dementia, palliative care, informal caregiving and supportive interventions.

Previous research suggests that possessing sufficient knowledge about dementia is essential for providing high‐quality care for individuals with dementia (Smyth et al. 2013). For example, a lack of knowledge and education among nurses has been observed to result in the use of physical force or chemical restraints to manage uncooperative patients (Jacobsen et al. 2017; Nilsson et al. 2016). Additionally, healthcare professionals with negative attitudes and perceptions may demonstrate a decreased commitment to providing optimal interventions (de Levante Raphael 2022). Furthermore, nurses who exhibit unfavourable attitudes towards individuals with dementia often experience heightened level of stress and increased turnover rate (Sung et al. 2005). To effectively tackle the challenges associated with dementia care, Zhao et al. underscore the importance of understanding nurses' knowledge and attitudes towards dementia (Zhao et al. 2022).

Several studies have examined nurses' knowledge and attitudes towards dementia, including nursing students and nurses in various units (Aljezawi et al. 2022; Romem et al. 2023). Findings show that while most nurses possess basic knowledge regarding dementia (Attard et al. 2020), there remains a significant gap in their level of understanding (Lin et al. 2018). Factors such as educational background, nursing experience, work unit and received training are associated with the level of dementia knowledge (Lin et al. 2018). Additionally, age and prior dementia care experience influence attitudes towards dementia care (Zhao et al. 2022). Variations in care for individuals with dementia across organisations are attributed to differing working conditions (Yaghmour 2022).

Chinese nurses hold a distinctive and crucial role in the field of Alzheimer's disease care. Given China's substantial population base and the accelerating aging process, the prevalence of Alzheimer's disease is increasing. As integral components of the healthcare system, Chinese nurses are directly engaged in the daily care of Alzheimer's patients. Their attitudes significantly influence not only the quality of patient care but also the psychological support and overall quality of life for both patients and their families. Wuxi is situated in the economically advanced Yangtze River Delta region of mainland China and exhibits specific characteristics regarding its level of economic development, allocation of medical resources and population structure, among other factors. Furthermore, Wuxi's healthcare system is relatively comprehensive, encompassing general hospitals, community hospitals and nursing homes, thereby offering a full spectrum of medical services for patients with Alzheimer's disease. The survey to understand the knowledge and attitudes of nurses in Wuxi area about Alzheimer's disease can provide a valuable resource for improving the care of Alzheimer's disease by geriatric specialist nurses.

## 3 Methods

### 3.1 Research Design

A cross‐sectional study.

### 3.2 Participants and Data Collection

Registered nurses from 5 hospitals, 8 community centers and 4 nursing homes in Wuxi, China, were recruited for this study through convenience sampling. The inclusion criteria for participants were as follows: (1) possession of a valid nursing qualification certificate, (2) engagement in geriatric nursing‐related work and (3) willingness to participate in the study. Exclusion criteria included nurses who had not been actively employed in the past 6 months due to reasons such as sick leave, maternity leave or further education.

This study utilised anonymized data collected through an online questionnaire distributed via the widely utilised questionnaire website in the Chinese Mainland, https://www.wjx.cn/, from January to March 2024. The questionnaire was disseminated to a WeChat group comprised of nursing managers in individual hospitals in Wuxi and participation was voluntary. Each IP address was limited to submitting only one questionnaire, with all questions being mandatory. Questionnaires exhibiting logical errors (e.g. impossible age), consistent filling patterns (e.g. selecting all first choices), or completed in less than 90 s or more than 6000 s were deemed invalid. The research team members checked all questionnaires' completeness, consistency and validity.

The sample size should be 5–10 times the number of items on the scale (Zhan et al. 2020). Considering a 10% rate of invalid questionnaires, a minimum of 167 participants was necessary for this study. Ultimately, 192 nurses participated in the survey, with 6 questionnaires excluded due to incompleteness. This resulted in a total of 186 valid questionnaires, yielding a completion rate of 96.88%.

### 3.3 Instruments

#### 3.3.1 General Information Questionnaire

General information questionnaire was created to gather information on participants' socio‐demographic data including gender: (Male/Female), age: (20–30/31–40/41–50/51 years and above), education: (Junior College/Bachelor/Master), Professional Title: (Junior/Intermediate/Senior), Workplace: (Hospital/Community Centre/Nursing Home), Work experience: (Under 2 years/2–5 years/6–10 years/11 years and above), Previous experience in ad care: (Yes/No), Previous training in ad care: (Yes/No) and Willingness to educate about ad: (Yes/No). These factors were selected based on previous research (Evripidou et al. 2019; Lin et al. 2019; Zhao et al. 2022).

#### 3.3.2 Alzheimer's Disease Knowledge Scale (ADKS)

The ADKS scale was chosen to evaluate knowledge levels due to its robust psychometric properties, evidenced by a Cronbach's alpha coefficient of 0.71, and its successful cultural adaptation within the Chinese context (Carpenter et al. 2009). The Chinese version was translated in 2013 by He Runlian et al., with a Cronbach's alpha coefficient of 0.756 (He et al. 2013). The scale consists of 30 true/false items distributed across 7 dimensions: risk factors (6 items); assessment and diagnosis (4 items); symptoms (4 items); course of the disease (4 items); life impact (3 items); nursing (5 items); and treatment and management (4 items). Each item is scored as 1 point, with the total score ranging from 0 to 30, reflecting the respondent's level of knowledge. A higher score indicates more knowledge.

#### 3.3.3 Dementia Attitudes Scale (DAS)

The DAS was selected to assess the attitudes of nurses towards individuals with dementia. This scale is grounded in a tripartite model of attitude, encompassing emotional, cognitive and behavioural components and is reported to possess robust psychometric properties, with a reliability coefficient ranging from 0.830 to 0.850 (O'Connor and McFadden 2010). In 2019, Li Huanli translated this scale into Chinese, with a reliability coefficient 0.787 (Li et al. 2019). Three common factors, accounting for 48.026% of the variance, were identified: dementia knowledge, positive social comfort and negative social comfort. The scale comprises 20 items that measure attitudes using a Likert scale, with total scores ranging from 20 to 140 points. Items 2, 6, 8, 9, 16 and 17 are reverse scored. A higher score indicates a more positive attitude towards patients with dementia.

### 3.4 Data Analysis

The data analysis was conducted using IBM SPSS version 27.0. Descriptive statistics, including means, standard deviations and percentages were calculated. Normality tests were performed on the data. Independent sample *t*‐tests and one‐way analysis of variance were utilised to examine the social demographic variables of nurses across different groups. The term “variables” encompasses all sociodemographic characteristics of the nursing staff, including gender, age, education, professional title, workplace, work experience, previous experience in ad care, previous training in ad care, willingness to educate about ad, each of which was incorporated into the data set. Independent sample *t*‐tests and one‐way analysis of variance were conducted. Given that the nurses participating in this study were drawn from hospitals, community settings and nursing homes, and acknowledging that different organisations may exert varying influences on the knowledge and attitudes of nurses, a variance inflation factor analysis was conducted to assess the presence of collinearity among the different organisational contexts. Factors identified as statistically significant in the univariate analysis were subsequently included in the multiple linear regression analysis (Lin et al. 2019). To identify the factors affecting nurses' knowledge and attitudes towards ad patients, multiple linear regression analysis was conducted, and R version 4.4.2 was utilised to create the forest plot. All statistical tests were two‐tailed, with a significance level set at 0.05.

### 3.5 Ethical Considerations

The study was approved by the hospital ethics committee. Participants engaged in the study voluntarily and anonymously. Before commencing the questionnaire, two inquiries were made to ascertain an individual's willingness to participate in the survey and to confirm their understanding of the survey's academic research nature. Only participants who provided explicit consent were included in the final analysis. We ensured that informed consent was obtained and documented through electronic signatures. The confidentiality of all collected data was maintained, with data being utilised solely for the purpose of this study.

## 4 Results

### 4.1 Participant Characteristics

A total of 186 nurses submitted valid responses in this study. Approximately 98.9% (*n* = 184) of the participants were female, and half were aged between 31 and 40 (*n* = 93, 50.0%). Nurses with a bachelor's degree were the largest group (*n* = 162, 87.1%), and more than half of the nurses had a junior professional title (*n* = 94, 50.5%). One hundred and eight participants work in hospitals (*n* = 58.1%). More than half of the participants had experience with ad care (*n* = 111, 59.7%). Most participants (*n* = 139, 74.7%) had prior training in ad caring. Table 1 showed the sociodemographic characteristics of nurses.

**TABLE 1:** The socio‐demographic data of nurses(*n* = 186).

Variables	Number	Percentage (%)
Gender		
Male	2	1.1
Female	184	98.9
Age		
20–30	76	40.9
31–40	93	50.0
41–50	16	8.6
51 years and above	1	0.5
Education		
Junior College	22	11.8
Bachelor	162	87.1
Master	2	1.1
Professional Title		
Junior	94	50.5
Intermediate	80	43.0
Senior	12	6.5
Workplace		
Hospital	108	58.1
Community Centre	68	36.6
Nursing Home	10	5.4
Work experience		
Under 2 years	17	9.1
2–5 years	33	17.7
6–10 years	53	28.5
11 years and above	83	44.6
Previous experience in AD care		
Yes	111	59.7
No	75	40.3
Previous training in ad care		
Yes	139	74.7
No	47	25.3
Willingness to educate about AD		
Yes	160	86.0
No	26	14.0

### 4.2 Knowledge and Attitude Towards Dementia of Nursing Staff

Table 2 showed the participants score of adKS measuring knowledge of dementia. The total possible adKS score is 30, with the average score for the participants being 20.82 ± 2.31. The correct response rates across the seven dimensions ranged from 55% to 85%. The dimensions ‘symptoms’ (55%) and ‘nursing’ (60%) had significantly lower correct response rates compared to other dimensions, while ‘treatment and management’ had the highest correct response rate at 85%. Table 3 displays the scores for nurses' attitudes towards dementia, as measured by the Dementia Attitudes Scale (DAS). The total DAS score is 140, with the average score for nurses being 86.23 ± 14.14 (approximately 61.6%).

**TABLE 2:** Dementia knowledge measured by the ADKS (*n* = 186).

Domains	ltems	Range of total score	Mean ± SD	Mean correct rate (%)
Risk factor	2,13,18,25,26,27	0–6	4.17 ± 0.91	69.5
Assessment and diagnosis	4,10,20,21	0–4	3.00 ± 0.55	75.0
Symptom	19,22,23,30	0–4	2.19 ± 0.83	54.8
Course of disease	3,8,14,17	0–4	3.12 ± 0.69	78.1
Life impact	1,11,28	0–3	1.94 ± 0.67	64.7
Caregiving	5,6,7,15,16	0–5	2.98 ± 1.00	59.6
Treatment and management	9,12,24,29	0–4	3.41 ± 0.71	85.2
Total knowledge mean score		0–30	20.82 ± 2.31	69.4

**TABLE 3:** Attitudes to dementia measured by the DAS (*n* = 186).

Domains	ltems	Range of total score	Mean ± SD	Percentage value (%)
Dementia knowledge	7,10,11,12,14,15,18,19,20	9–63	38.71 ± 8.37	61.4
Positive social comfort	1,3,4,5,13	5–35	18.59 ± 4.43	53.1
Negative social comfort	2,6,8,9,16,17	6–42	28.92 ± 5.96	68.9
Total attitude mean score		20–140	86.23 ± 14.14	61.6

### 4.3 Influencing Factors of the Knowledge and Attitude of Nursing Staff

**FIGURE 1:**
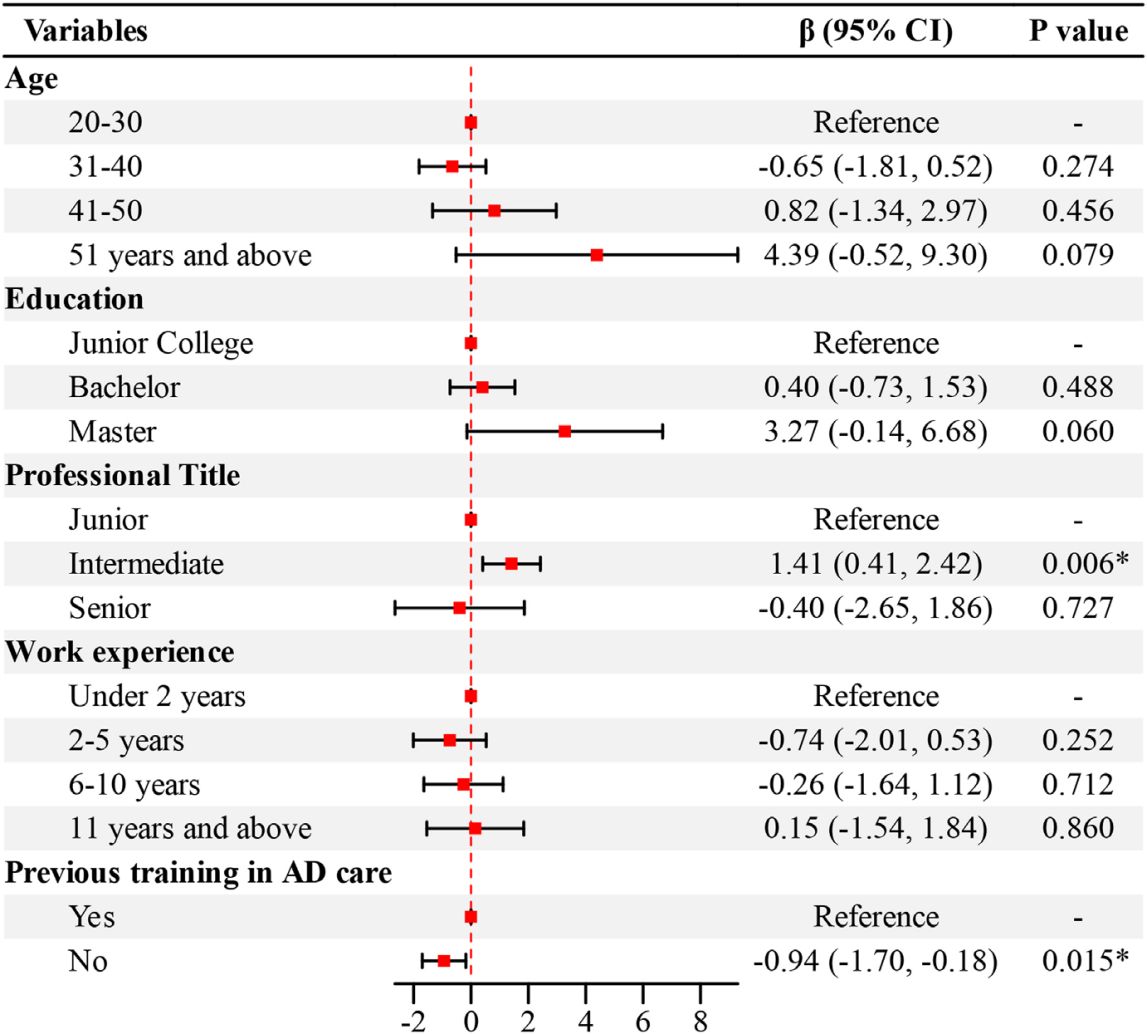
Linear regression forest plots of ADKS related factors.

**FIGURE 2:**
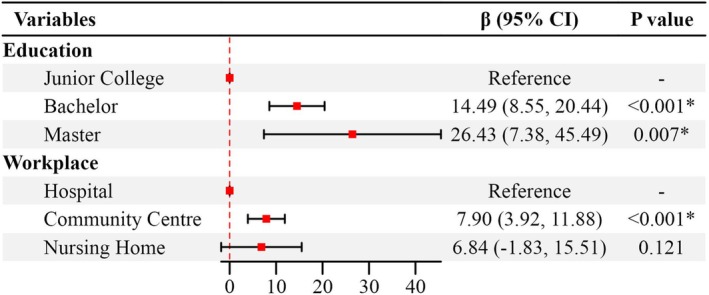
Linear regression forest plots of DAS related factors.

Table 4 showed significant differences in adKS scores among nurses based on age, education, professional title, work experience and previous training in ad care(*p* < 0.05). DAS scores varied significantly based on education and workplace (*p* < 0.05). Variance inflation factor analysis showed that there was no collinearity between different workplaces (VIF = 1.03). Dummy variables were set for categorical variables: ‘20–30’ as the reference group for age, ‘junior college’ for education, ‘junior’ for professional title, ‘hospital’ for workplace, ‘under 2 years’ for work experience and ‘yes’ for previous training in ad care. Multiple linear regression with adKS total score as the dependent variable and age, education, professional title, work experience and previous training in ad care as independent variables (Figure 1). Multiple linear regression with DAS total score as the dependent variable and education and workplace as independent variables (Figure 2). The linear regression model revealed that professional title and prior training in ad care were independent factors influencing dementia knowledge among nurses (*p* < 0.05), explaining approximately 14.4% of the variance in ADKS scores. Education and workplace were independent factors affecting attitudes towards dementia (*p* < 0.05), accounting for about 15.7% of the variance in DAS scores (Table 5).

**TABLE 4:** Influencing factors of the total score of ADKS and DAS (*n* = 186).

General information	Unweighted number	adKS *x* ± *s*	*F*/*t*‐value	*p*	DAS *x* ± s	*F*/*t*‐value	*p*
Gender							
Male	2	18.50 ± 0.71	−1.431	0.154	84.00 ± 1.41	0.223	0.824
Female	184	20.84 ± 2.31			86.25 ± 14.21		
Age							
20–30	76	20.29 ± 2.19	3.746	0.012*	84.84 ± 13.60	1.295	0.278
31–40	93	21.04 ± 2.29			88.06 ± 14.42		
41–50	16	21.75 ± 2.38			82.81 ± 14.55		
51 years and above	1	25			75		
Education							
Junior College	22	19.55 ± 1.63	4.115	0.018*	74.18 ± 12.34	10.395	< 0.001*
Bachelor	162	20.98 ± 2.35			87.73 ± 13.64		
Master	2	22.00 ± 0.00			96.50 ± 4.95		
Professional Title							
Junior	94	20.06 ± 2.12	11.346	< 0.001*	84.38 ± 12.96	2.227	0.111
Intermediate	80	21.63 ± 2.20			88.73 ± 15.41		
Senior	12	21.33 ± 2.67			84.00 ± 12.42		
Workplace							
Hospital	108	21.06 ± 2.37	2.460	0.088	83.44 ± 14.57	5.893	0.003*
Community Centre	68	20.63 ± 2.21			90.75 ± 12.71		
Nursing Home	10	19.50 ± 1.96			85.60 ± 11.48		
Work experience							
Under 2 years	17	20.47 ± 2.00	4.537	0.004*	81.12 ± 16.12	2.634	0.051
2–5 years	33	19.73 ± 2.04			82.70 ± 13.16		
6–10 years	53	20.72 ± 2.20			89.81 ± 12.33		
11 years and above	83	21.39 ± 2.39			86.39 ± 14.74		
Previous experience in AD care							
Yes	111	21.08 ± 2.32	1.910	0.058	87.86 ± 12.91	1.927	0.055
No	75	20.43 ± 2.24			83.81 ± 15.55		
Previous training in AD care							
Yes	139	21.07 ± 2.34	2.629	0.009*	87.35 ± 13.80	1.882	0.061
No	47	20.06 ± 2.07			82.89 ± 14.75		
Willingness to educate about AD							
Yes	160	20.74 ± 2.29	−1.077	0.283	86.58 ± 14.63	0.850	0.396
No	26	21.27 ± 2.43			84.04 ± 10.54		

*Note:* Asterisks (*) indicate statistical significance.

**TABLE 5:** Multiple regression analysis for the ADKS and DAS (*n* = 186).

Independent variables	*B*	SE	*β*	*t*‐value	*p*	VIF	95% confidence interval	
ADKS (Constant)	20.46	0.64		32.23	< 0.001		19.21	21.71
Age						3.58		
20–30	—	—	—	—	—	—	—	—
31–40	−0.65	0.59	−0.14	−1.097	0.274		−1.81	0.52
41–50	0.82	1.09	0.10	0.747	0.456		−1.34	2.97
51 years and above	4.39	2.49	0.14	1.765	0.079		−0.52	9.30
Education						1.25		
Junior College	—	—	—	—	—	—	—	—
Bachelor	0.40	0.57	0.06	0.696	0.488		−0.73	1.53
Master	3.27	1.73	0.15	1.891	0.060		−0.14	6.68
Professional Title						2.93		
Junior	—	—	—	—	—	—	—	—
Intermediate	1.41	0.51	0.30	2.775	0.006*		0.41	2.42
Senior	−0.40	1.14	−0.04	−0.350	0.727		−2.65	1.86
Work experience						2.44		
Under 2 years	—	—	—	—	—	—	—	—
2–5 years	−0.74	0.64	−0.12	−1.149	0.252		−2.01	0.53
6–10 years	−0.26	0.70	−0.05	−0.370	0.712		−1.64	1.12
11 years and above	0.15	0.86	0.03	0.176	0.860		−1.54	1.84
Previous training in AD care						1.03		
Yes	—	—	—	—	—	—	—	—
No	−0.94	0.39	−0.18	−2.450	0.015*		−1.70	−0.18
*R* = 0.442 *R* ^2^ = 0.195 Adjusted *R* ^2^ = 0.144 *F* = 3.831 (*p* < 0.001)								
DAS (Constant)	70.07	3.01		23.29	< 0.001		64.13	76.00
Education						1.03		
Junior College	—	—	—	—	—			
Bachelor	14.49	3.02	0.35	4.804	< 0.001*		8.55	20.44
Master	26.43	9.66	0.19	2.737	0.007*		7.38	45.49
Workplace						1.03		
Hospital	—	—	—	—	—			
Community Centre	7.90	2.02	0.27	3.914	< 0.001*		3.92	11.88
Nursing Home	6.84	4.39	0.11	1.558	0.121		−1.83	15.51
*R* = 0.418 *R* ^2^ = 0.175 Adjusted *R* ^2^ = 0.157 *F* = 9.604 (*p* < 0.001)								

*Note:* Asterisks (*) indicate statistical significance.

## 5 Discussion

### 5.1 Current Situation of Knowledge and Attitude Towards Dementia Among Nursing Staff

#### 5.1.1 Current Situation of Knowledge of Dementia Among Nursing Staff

The level of ad knowledge among nursing staff in Wuxi, China was found to be relatively low, with the overall correct response being only 69%. This finding aligns with previous research indicating that nursing staff in China generally have insufficient dementia knowledge (Lin et al. 2019; Wang et al. 2018). Compared with the previous studies, the Wuxi ad knowledge level of nurses (Mean ± SD: 20.41 ± 2.94) is similar to that among healthcare professionals in India (Mean ± SD: 19.2 ± 3.1) but lower than the level observed among healthcare professionals in Australia (Mean ± SD: 23.60 ± 3.26) (Singh et al. 2022; Smyth et al. 2013). Significant knowledge deficits were observed in the dimensions of ‘Symptoms’ (correct rate 55%) and ‘Caregiving’ (correct rate 60%). Although 88.2% of the nursing staff held a bachelor's degree or higher, their scores in Alzheimer's disease symptoms and caregiving knowledge were notably low. This may be attributed to inadequate education on Alzheimer's disease during their academic training (Zhao et al. 2022). This is consistent with the findings of Wang et al. on the mastery of ad knowledge among community health professionals. They underscore the need for ongoing education and professional development in dementia care, including the implementation of Alzheimer's disease‐related courses, knowledge lectures and online resources to enhance the awareness and expertise of in‐service nurses (Wang et al. 2018).

#### 5.1.2 Current Situation of Attitude Towards Dementia Among Nursing Staff

In this study nursing staff attitudes towards Alzheimer's disease are averaged 86.23 ± 14.14. This finding was higher than the positive attitude levels of nurses in Jordan (Mean ± SD: 83.8 ± 14.4) (Aljezawi 2021). This finding is in contrast to the conclusions of the review, which highlighted the prevalence of negative attitudes among nursing staff (Zhao et al. 2022). The relatively high positive attitude scores of nurses in this study may have benefited from a series of effective professional training. Since 2021, Wuxi Nursing Association has organised geriatric professional nurse training every year, covering clinical practice and theoretical training, which makes nursing staff more confident and more positive in the face of Alzheimer's disease, a common disease in the elderly. The negative attitudes of nurses identified in previous reviews are not without justification. A significant contributing factor is the stigma associated with individuals with dementia. Individuals with Alzheimer's disease face more stigma due to shared symptoms with schizophrenia, like agitation, depression and delusions (Stites et al. 2018). Media portrayals often perpetuate these stereotypes by depicting elderly individuals with dementia as irritable and uncontrollable (Low and Purwaningrum 2020). Consequently, such representations have subtly influenced the perceptions of nurses regarding individuals with dementia. Even when nurses have received professional training, exposure to such societal views can lead to the development of negative emotions. Additionally, the challenging and high‐pressure nature of caring for individuals with Alzheimer's disease provides fertile ground for negative attitudes to emerge. The unpredictable nature of the disease, coupled with the continuous decline in patients' cognitive and behavioural abilities, requires nurses to expend considerable effort in caregiving, often with limited perceived effectiveness, leading to accumulated frustration (Kang and Hur 2021). Consequently, alongside the psychological burden of stigma, negative attitudes are understandably prevalent among nurses. This prevalence may account for the predominance of negative attitudes reported in previous studies, contrasting with the findings of the current investigation.

### 5.2 Factors Affecting Nursing Staff Knowledge of People With Dementia

In this study, we found that professional title and previous training in ad were factors influencing nurses' dementia knowledge. Nurses with intermediate professional titles exhibited higher adKS scores compared to those with junior and senior titles, suggesting a greater awareness of ad among the former group. In China, the evaluation of professional titles assesses nurses' overall quality across various dimensions, which facilitates a transition from quantitative to qualitative improvements in nursing competencies. This, in turn, enhances the quality of nursing services. Nurses holding intermediate titles often have extensive professional knowledge and play a crucial role in dementia care. Conversely, the limited knowledge among nurses with junior titles may be due to the lack of dedicated dementia‐themed courses in nursing curricula and the restricted availability of dementia‐specific educational programs (Zhao et al. 2022). Similarly, the level of dementia education within pre‐registration care programs in the UK is insufficient. Tuffour et al. emphasise that access to high‐quality dementia care is contingent upon comprehensive pre‐registration nursing education (Tuffour and Ganga 2024). They advocate for the establishment of a distinct pre‐registration branch dedicated to dementia specialist nurse education. In this context, China could benefit from adopting similar measures, such as incorporating a dedicated dementia course into the nursing education curriculum and integrating assessments of dementia‐related knowledge and skills into the nursing qualification examination. Although no studies have specifically examined the impact of senior titles on knowledge levels, it has been observed that years of experience do not necessarily correlate with advanced competence (Sevilla Guerra et al. 2018). Pilcher (2010) have similarly concluded that nurses' interest in education diminishes with increasing seniority. However, in the contemporary context, characterised by demographic shifts and an aging population impacting professional groups such as nurses, it is crucial to effectively motivate and foster the development of older employees. Consequently, it is imperative to develop targeted dementia education and care policies to address the knowledge and training gaps related to dementia among medical staff across various professional levels. Such policies would enable nurses with different professional titles to acquire the specific skills necessary for managing ad.

This study demonstrated that nursing staff who had undergone training related to Alzheimer's disease knowledge or skills exhibited a higher adKS score (21.07 ± 2.34) compared to those who had not received such training, whose adKS score was comparatively lower (20.06 ± 2.07). The difference between the scores of the two groups was statistically significant (*p* < 0.05). These findings are consistent with those reported by Lin et al., indicating a correlation between training and knowledge acquisition (Lin et al. 2018). The findings indicated that caregivers who underwent training on AD demonstrated a superior understanding of AD‐related knowledge, corroborating the results of the study conducted by Wang et al. (2017). In Wuxi, annual academic activities focused on Alzheimer's disease in the elderly are organised, and medical personnel receive professional credits for participating in these training sessions. Furthermore, geriatric professional nurse training is held annually in Wuxi to improve the quality of geriatric nursing care. Previous research has demonstrated the effectiveness of dementia education program that incorporates WeChat‐based learning interactions in enhancing nurses' attitudes, knowledge and intentions regarding dementia care (Wang et al. 2017). Moreover, Japanese researchers employed virtual reality interventions to deliver dementia care education to emergency nurses, resulting in a significant enhancement of the nurses' dementia care competencies (Yamaguchi et al. 2022). Future developments in training methodologies, particularly the incorporation of virtual reality simulation training, have the potential to further augment the empathy and practical skills of nursing personnel in the management of patients with dementia.

### 5.3 Factors Affecting Nursing Staff Attitude Towards People With Dementia

We found that education background and workplace influenced nurses' attitudes towards dementia. This study demonstrated that nurses with higher levels of education exhibited more positive attitudes, aligning with findings from research conducted in Macau (Wong et al. 2024). A study examining nursing knowledge and attitudes in long‐term care institutions in Japan revealed that nurses with advanced educational qualifications had significantly higher dementia attitude scores (Nakanishi and Miyamoto 2016). Previous research has indicated that nurses with higher qualifications are more inclined to engage in continuing education training (Bartosiewicz et al. 2019), and another study has established a positive correlation between learning behaviours and attitudes towards dementia care (Smyth et al. 2013). Highly educated nurses typically exhibit a positive disposition towards continuing education and demonstrate a willingness to enhance their competencies in dementia care through participation in relevant training programs and seminars. This commitment enables them to update their knowledge base and sustain a constructive professional attitude. Furthermore, nurses possessing advanced educational qualifications often benefit from more extensive theoretical and clinical training, which may contribute to a deeper understanding and greater empathy for individuals with dementia. Highly qualified nurses are more adept at interpreting patient behaviours through the lens of dementia pathology, acknowledging these behaviours as symptomatic manifestations rather than reflections of individual character flaws. Conversely, nurses with lower qualifications may exhibit a greater need for ongoing education. Consequently, future research should investigate whether the attitudes of both highly and less educated nurses improve significantly following identical training in dementia care, as well as identify the factors influencing these attitudinal changes.

Our study further demonstrated that community nurses exhibited more positive attitudes compared with their counterparts in hospitals and nursing homes, aligning with the findings of Evripidou et al. (2019). Additionally, research indicates that older community healthcare professionals tend to display a more favourable attitude towards individuals with dementia (Wang et al. 2018). These attitudes are critical determinants of the quality of dementia care, as they influence the type and extent of health information communicated to patients with dementia and their family caregivers. Blaser and Berset (2019) also established a strong correlation between the nursing environment and attitude scores. In the acute hospital setting, individuals with dementia often receive lower prioritisation. Medical personnel typically prioritise patients presenting with acute conditions, such as cardiovascular and cerebrovascular diseases, fractures and similar emergencies, resulting in the frequent neglect of those with cognitive impairments. Some nurses in acute care facilities have reported reluctance to provide care to elderly patients (Deasey et al. 2014). Furthermore, factors such as the supportive environment within the workplace, patient volume and overall workload significantly impact the mental health and attitudes of nursing staff. Nurses operating in high‐workload or low‐resource settings within acute care hospitals are susceptible to burnout, potentially influencing their attitudes towards individuals with dementia (Keogh et al. 2020). It is imperative to collaborate with educational institutions to implement an extensive and comprehensive educational program for staff engaged in direct patient care. This program should encompass detailed information on dementia severity and equip participants with the specialised skills necessary to deliver high‐quality care to dementia patients in acute care environments.

## 6 Limitations

The present study was conducted in Jiangsu; however, variations in cultural and economic contexts, as well as differences in dementia incidence, may lead to discrepancies in the knowledge and attitudes of nurses in other countries or regions compared to our findings. Consequently, it is imperative to undertake studies with larger sample sizes in the future to examine the current state of nurses' knowledge and attitudes across diverse regions and to analyse the factors influencing these aspects. Furthermore, given the limitations inherent in a cross‐sectional study design, future research should employ longitudinal studies to investigate causal relationships. Such studies would enable the development of targeted interventions based on the findings from large‐sample longitudinal research, aimed at enhancing the knowledge and attitudes of nurses.

## 7 Conclusion

With the accelerating global aging, ad has emerged as a significant public health challenge.

In China, the rapidly expanding elderly population faces economic constraints that often limit their access to high‐quality care, exacerbating social and familial burdens. Nurses play a crucial role in the care of patients with ad and having comprehensive knowledge of the disease is essential for providing high‐quality care. Although the study primarily concentrates on the Wuxi area, it holds potential reference value for nurse education, clinical practice and disease management on a global scale through comparative analysis with international research. The implementation of systematic training programs aimed at enhancing the knowledge and attitudes of nursing staff can lead to improved patient outcomes and alleviate societal pressures. Furthermore, promoting the professional development of nursing staff is essential for addressing the challenges associated with an aging population in China.

## Author Contributions

R.W., Y.G. and H.Y. performed data analysis; R.W. drafted the original manuscript. S.G., Y.C. and X.W. significantly provided feedback to the manuscript. All listed authors are to have contributed to the manuscript substantially, agreed to the order in which the author names appear and agreed to the final submitted version.

## Ethics Statement

The Ethics Committee of (omitted for double‐anonymized peer review) approved this study (Ethics Number: omitted for double‐anonymized peer review). Written informed consent form was obtained from all participants.

## Conflicts of Interest

The authors declare no conflicts of interest.

## Public Contribution

Registered nurses from 5 hospitals, 8 community centers and 4 nursing homes in Wuxi participated in this study.

## Data Availability

Please get in touch with the corresponding author if you would like the data supporting the findings of this study. The data are not publicly available due to privacy or ethical restrictions.
